# Probiotic consortium modulating the gut microbiota composition and function of sterile Mediterranean fruit flies

**DOI:** 10.1038/s41598-023-50679-z

**Published:** 2024-01-11

**Authors:** Hamden Haytham, Charaabi Kamel, Djobbi Wafa, Fadhel Salma, Bel Mokhtar Naima, Tsiamis George, Cherif Ameur, Meriem Msaad Guerfali

**Affiliations:** 1 Laboratory of Biotechnology and Nuclear Technologies, LR16CNSTN01, National Centre of Nuclear Sciences and Technologies, Sidi Thabet, Tunisia; 2 Laboratory of Systems Microbiology and Applied Genomics, Department of Sustainable Agriculture, University of Patras, Agrinio, Greece; 3 Laboratory of Innovative Technology, National School of Applied Sciences of Tangier, Abdelmalek Essâadi University, Tétouan, Morocco; 4 Higher Institute of Biotechnology Sidi Thabet, BVBGR-LR11ES31, University of Manouba, Biotechpole Sidi Thabet, Ariana, Tunisia

**Keywords:** Environmental microbiology, Computational biology and bioinformatics, Sequence annotation

## Abstract

The sterile insect technique (SIT) remains a successful approach in managing pest insects. However, the long-term mass rearing and sterilizing radiation associated with SIT have been observed to induce physiological and ecological fitness decline in target insects. This decline may be attributed to various factors, including commensal microbiota dysbiosis, selection procedures, loss of heterozygosity, and other complex interactions.. There is evidence that the bacterial symbiont of insects may play critical roles in digestion, development, reproduction, and behavior. Probiotics are an increasingly common approach for restoring the intestinal microbiota structure and fitness parameters of sterile insects, particularly in the Vienna 8 genetic sexing strain (V8-GSS) of the Mediterranean fruit fly (medfly), *Ceratitis capitata*. Here, we explore the influence of the previously isolated bacterial strain, *Lactococcus lactis*, *Enterobacter* sp., and *Klebsiella oxytoca*, administration as probiotic consortia (LEK-PC) to the larvae and/or adult diet over the course of 20 rearing generations on fitness parameters. The experiment was carried out in four colonies: a control colony (C), one to which probiotics were not added, one to which probiotics were added to the larval medium (L+), one to which probiotics were added to the adult medium (A+), and one to which probiotics were added to both the larval and adult mediums (AL+). Emergence, flight ability, survival under stress conditions, and mating competitiveness, were all significantly improved by the LEK-PC treatment independently of the administration stage. The intestinal microbiota structure of various medfly V8-GSS colonies also underwent a significant shift, despite the fact that the core microbial community was unaffected by the LEK-PC administration stage, according to 16S metagenomics sequencing. Comparison of the metabolic function prediction and associated carbohydrate enzymes among colonies treated with “LEK-PC” showed an enrichment of metabolic functions related to carbohydrates, amino acids, cofactors, and vitamins metabolism, as well as, glycoside hydrolase enzymes in the AL+ colony compared to the control. This study enriches the knowledge regarding the benefits of probiotic treatment to modulate and restore the intestinal microbiota of *C. capitata* sterile males for a better effectiveness of the SIT.

## Introduction

Insects’ intestinal tracts are home to a variety of microorganisms. This relationship significantly influences diverse aspects of insect biology, such as nutrition and digestion, development, physiology, immune defense, and pesticide resistance, allowing insects to adapt to a variety of environmental and dietary conditions^1–4^.

Studies of these symbiotic associates contribute to our understanding to insect physiology and help to shape and improve the effectiveness of control and prevention strategies^1,2^. The sterile insect technique (SIT) is one of these techniques that efficiently manage insect pest populations when used as a component of an area-wide integrated pest management strategy (AW-IPM)^5^. SIT is a highly target-specific and environmentally friendly approach that involves mass-producing, sexually sterilizing, and then releasing the target pest insect ^6^. When the the sterile male flies are released they will likely mate with wild females, and produce no viable offspring, eventually causing the population to be suppressed^7^. Howerver, it has been reported that irradiation can compromise the structure of the insect gut community, leading to a decline in insect fitness, particularly in terms of mating competitiveness, flight performance, and survival^8,9^. Beyond irradiation, additional factors, such as selection procedures and loss of heterozygosity, may also contribute to the observed decline in insect fitness. Moreover, the long-term artificial rearing of insects has been shown to negatively impact the structure of the gut microbiota by reducing both bacterial and functional diversity^10,11^.

The use of probiotics as food additives is one method for restoring the commensal microbiome groups in various SIT manifestations of dysbiosis^9,12–21^. In this context, probiotics have received more attention recently in SIT programs using insect mass-rearing facilities in this context. Probiotics are defined as "live microorganisms that, when administered in adequate amounts confer a health benefit on the host"^22^.

The Mediterranean fruit fly (Medfly), *Ceratitis capitata* (Diptera: Tephritidae), is one of the most damaging insect pests, causing significant economic losses to a diverse agricultural crops^23–25^. It served as a model for the development of SIT programs and the potential use of the intestinal symbionts as probiotics during tephritid mass-rearing protocols^26^. The medfly’s microbial symbionts have been extensively studied particularly with modern molecular tools. Despite factors influencing the gut-associated microbial community such as environmental habitat, life stage, diet composition, rearing conditions, and strain, Enterobacteriaceae were found to predominate in the medfly gut using both conventional culture-dependent methods and high-throughput DNA sequencing technologies. *Klebsiella* and *Enterobacter* species predominated in many laboratory and wild medfly populations, followed by *Citrobacter, Providencia, Proteus, Pantoea*, etc., and possibly pathogenic bacteria like *Pseudomonas, Serratia*, and *Morganella*^8,11,26–39^. This gut core microbiome composition is typically present throughout all the fly’s life cycle stages and is vertically transmitted from parent to offspring. By obtaining and absorbing nitrogen and carbohydrate, it is essential for insect nutrition intake as well as for longevity, reproduction, and immunity^1,27,40–44^.

Previous studies have used single or multiple strains of *Klebsiella* and *Enterobacter* dominant bacteria in the diet of GSS medfly laboratory strains as larvae or adults^8,12–17^. They were chosen as probiotics based on the characterization of “in vivo” ecological fitness parameters. As a result, probiotic feeding beneficially affects the biological quality and fitness parameters of the sterile released males, including mating competitiveness, longevity, pupal weight, morphometric traits, and development duration^12–17^. However, a probiotic selection approach that combined “in vitro” and “in vivo” characterization was used to choose *Enterobacter* sp, *Lactococcus lactis*, and *Klebsiella oxytoca* as potential probiotic candidates to integrate into the diet of medfly larvae^15^. More recently, Savio et al.^45^ have drawn a general workflow and screening techniques to characterize probiotic potential strains for insect mass rearing. Moreover, advances in high-throughput technology have shifted attention to a novel approach for identifying a new array of potential probiotic strains from insect microbiomes through a metagenomic approach and a metabolic function prediction^46,47^.

Here we investigated the effect of probiotic consortia administration to V8-GSS medfly larvae and/or adult stages across 20 rearing generations, on different quality control parameters of insects. Furthermore, using high-throughput illumina sequencing of 16S rRNA gene, we also aimed to profile the changes in sterile male bacterial microbiota structure and metabolic activity in response to the probiotic feeding life stage administration. The findings in this study will contribute to a better understanding of the diversity and dynamics of the medfly gut microbiota after probiotic feeding and will provide improved strategies for modulating microbial structure within medfly sterile males towards a personalized population.

## Results

### Quality control parameters across LEK-PC treatment

**Figure 1:**
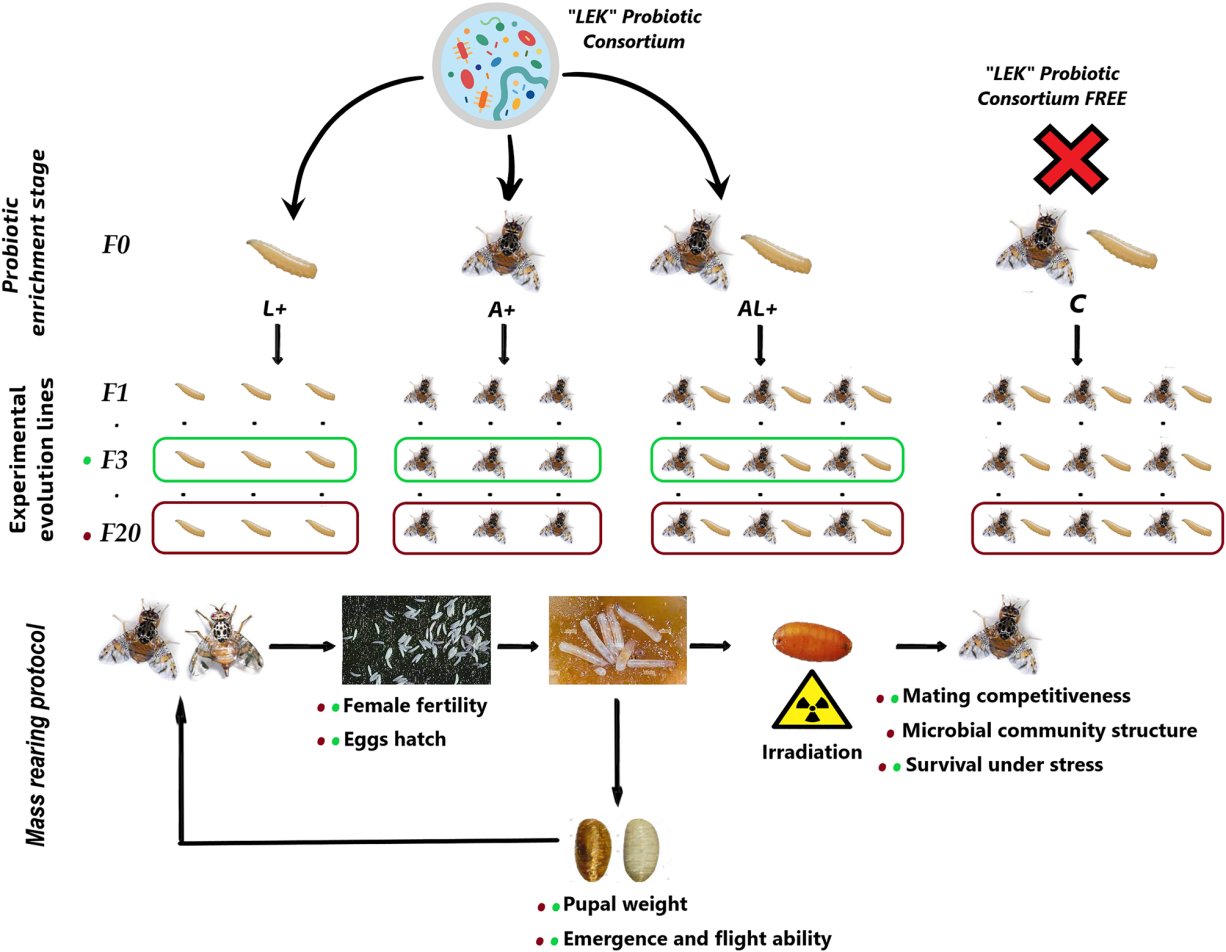
Schematic illustration depicting the LEK-PC feeding medfly colonies and their maintenance during 20 generations.

The Medfly colonies enriched with LEK-PC in the adult diet (A+), larval diet (L+), and both (AL+), as well as the control colony (C), were compared for quality control (QC) parameters at the F3 and F20 generations, as illustrated in Fig. 1 (refer to the “Materials and methods” section for details).

#### Effect of LEK-PC on pupal weight

The results revealed no significant differences between the tested colonies on male and female pupal weight at the 3rd generation (ANOVA: *F *_*(3, 8)*_ = 2.613, *P* = 0.123; ANOVA: *F *_*(3, 8)*_ = 1.091, *P* = 0.407, respectively) and the 20th generation (ANOVA: F _(3, 8)_ = 0.947, P = 0.462; ANOVA: F _(3, 8)_ = 1.903, P = 0.207, respectively) (Table 1). However, male pupal weight in L+ colony was significantly improved after “LEK-PC” treatment at the F3 generation compared to the F20 generation (t = 4.57, df = 2, P < 0.05) (Table 1).

**Table 1:** Quality control parameters recorded of the LEK-PC feeding medfly colonies at the 3rd and 20th rearing generation.

	Generations										Statistics (p-value)**			
	F3					F20								
	C	A+	L+	AL+	Stat*	C	A+	L+	AL+	Stat*	C	A+	L+	AL+
Male pupal weight (mg)	8.27 ± 0.02	8.33 ± 0.02	8.37 ± 0.04	8.35 ± 0.05	P = 0.123	8.33 ± 0.08	8.57 ± 0.13	8.65 ± 0.08	8.48 ± 0.2	P = 0.462	0.46	0.17	** < 0.05**	0.58
Female pupal weight (mg)	8.34 ± 0.06	8.52 ± 0.1	8.5 ± 0.06	8.44 ± 0.06	P = 0.40	8.49 ± 0.05	8.66 ± 0.07	8.6 ± 0.03	8.59 ± 0.02	P = 0.207	0.06	0.47	0.33	0.09
Survival under stress (%)	73.33 ± 2.98	73.33 ± 2.98	77.33 ± 6.18	78.66 ± 2.4	P = 0.96; P = 0.14	74.93 ± 0.68ab	80.76 ± 1.76bc	82 ± 2c	81.99 ± 1.33c	AL+ vs C : P < 0.05; P = 0.082	0.614	** < 0.05**	0.49	0.272
♀ emergence (%)	74 ± 4.16	76 ± 2.3	82 ± 1.15	982 ± 2	P = 0.287	74.66 ± 2.66	78 ± 3.46	85.33 ± 1.76	82.66 ± 2.4	P = 0.089	0.89	0.225	0.37	0.825
♂ emergence (%)	78 ± 2	80.66 ± 1.76	84 ± 3.05	82.66 ± 1.33	AL+ vs C : P < 0.05	79.33 ± 0.66	81.33 ± 1.76	85.33 ± 2.4	84	L+ vs C : P < 0.05P = 0.082	0.422	0.867	0.821	0.422
Sterile ♂ emergence (%)	71.33 ± 0.66	76.66 ± 1.33	81.33 ± 0.66	78.66 ± 1.76	L+ vs C : P < 0.01	73.33 ± 1.76	79.33 ± 1.33	84.66 ± 2.4	81.33 ± 2.4	P = 0.063	0.579	0.183	0.337	0.057
♀ flight ability (%)	70 ± 5.77	71.33 ± 2.66	78.66 ± 0.66	77.33 ± 0.66	P = 0.226	70.66 ± 1.76	72.66 ± 4.66	81.33 ± 1.76	78.66 ± 1.76	AL+ vs C : P < 0.01	0.913	0.634	0.269	0.528
♂ flight ability (%)	73.33 ± 1.76	76 ± 2	82 ± 3.05	80.66 ± 1.33	P = 0.066	74 ± 2	76.66 ± 2.66	84 ± 3.05	81.33 ± 1.33	L+ vs A+ : P < 0.05	0.422	0.884	0.7	0.666
Sterile ♂ flight ability (%)	67.33 ± 2.66	72 ± 1.15	80 ± 1.15	76 ± 3.46	L+ vs C : P < 0.01	67.33 ± 1.76	74 ± 1.15	82 ± 1.15	79.33 ± 2.4	L+ vs C : P < 0.01	0.439	0.25	0.477	** < 0.05**
Eggs/female/day	21.16 ± 2.35	23.18 ± 4.06	22.46 ± 1.46	27.49 ± 4.59	P = 0.597	20.53 ± 1.88	22.45 ± 1.8	23.45 ± 1.91	27.34 ± 1.03	P = 0.104	0.845	0.877	0.703	0.976
Egg hatch (%)	72.33 ± 2.33	71 ± 3.46	77 ± 1	74.66 ± 0.89	P = 0.297	73 ± 3.05	73.33 ± 3.28	77.66 ± 1.66	76.33 ± 3.28	P = 0.559	0.87	0.606	0.748	0.649

For each test, means ± standard error (*One way ANOVA test followed by Sidak’s post hoc test in each rearing generation, P < 0.05; **Student t-test between the two rearing generation F3 and F20 for each colony, P < 0.05).

Significant values are in bold.

#### Effect of LEK-PC on survival under stress

No statistical significance between colonies was detected in the survival of sterile males under food and water starvation at the 3rd generation (ANOVA: *F* (3, 16) = 0.831, *P* = 0.496) (Table 1). In contrast, at the 20th generation, the survival of sterile males under stress significantly improved after the “LEK-PC” treatment for the AL+ and L+ colonies compared to the control (P < 0.05), as well as between the F3 and F20 generations for the A+ sterile males (*t* = 2.714, df = 8, P < 0.05) (Table 1).

#### Effect of LEK-PC on emergence and flight ability

Male and female emergence and flight ability were not influenced by the “LEK-PC” treatment. Indeed, no significant difference was recorded between colonies at the F3 and F20 generations as well as between each colony across the two rearing generations (Table1). However, for sterile males, adult emergence was significantly influenced by the LEK-PC treatment in the AL+ colony at the F3 generation compared to the control (P < 0.05), and in the L+ colony at the F3 generation (P < 0.01) and F20 generation (P < 0.05) compared to the control. Flight ability also varied for sterile males at the 3rd generation in the L+ colony compared to the control (P < 0.01) and at the 20th generation in the AL+ and L+ colonies compared to the control (P < 0.01), and between the A+ and L+ colonies (P < 0.05). Significant improvement in the flight ability of AL+ sterile males was also found between the two rearing generations (t = 5, df = 2, P < 0.05) (Table 1).

#### Effect of LEK-PC on female fertility

The results of female fertility showed no significant effect between colonies after LEK-PC treatment on egg female productivity per day at the 3rd (ANOVA: *F*_(3, 8)_ = 0.663, *P* = 0.597) and 20th generation (ANOVA: *F*_(3, 8)_ = 2.858, *P* = 0.104), as well as on egg hatching percentage at the 3th (ANOVA: *F*_(3, 8)_ = 1.455, *P* = 0.297) and 20th generation (ANOVA: *F*_(3, 8)_ = 0.735, *P* = 0.559). No significant difference was also recorded for each colony across the assessed rearing generations on female fertility tests (Table 1).

#### Effect of LEK-PC on mating competitiveness

**Figure 2:**
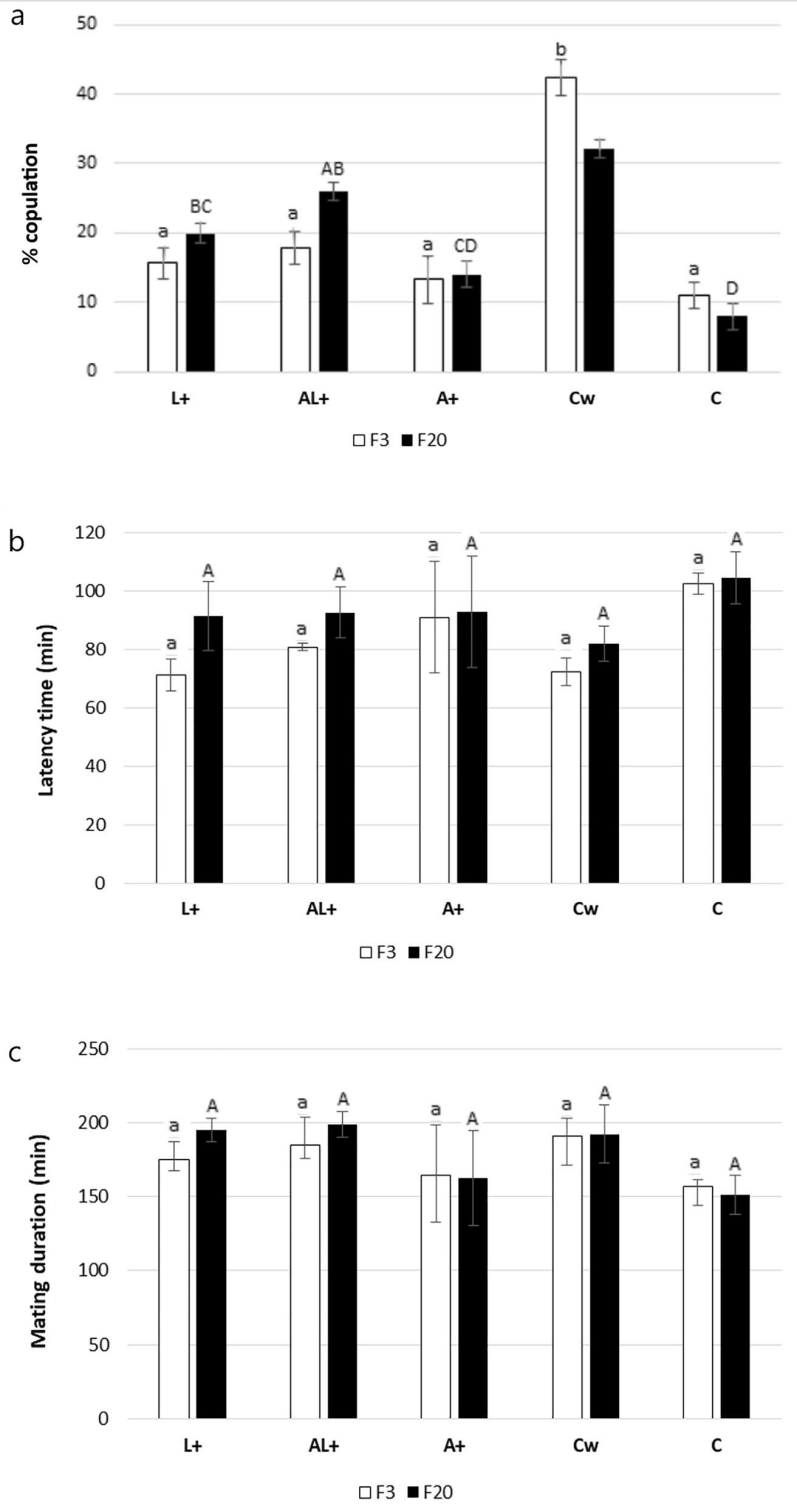
Effect of LEK-PC treatment on mating competitiveness between *C. capitata* sterile males colonies. Proportion of copulation (**a**), latency time (**b**) and mating duration (**c**) during the F3 and F20 generations. Different capital letters indicate significant differences among *C.capitata* colonies within F20 generation (One-way ANOVA test, P < 0.05). Different lower letters indicate significant differences among *C.capitata* colonies within F3 generation (One-way ANOVA test, P < 0.05).

The mating competitiveness test between A+, L+, AL+, C sterile males, and wildish male (Cw) for wildish female copulation revealed significant frequent mating pairs between wildish males with wildish females at the 3rd generation compared to the treated groups (ANOVA: F _(4, 10)_ = 23.86, P < 0.0001) (Fig. 2a). After 20 rearing generations, the results suggested a significant effect of LEK-PC treatment to improve L+ and AL+ sterile male mating competitiveness compared to control (ANOVA: F _(4, 10)_ = 36.3, P < 0.0001) (Fig. 2a). However, no significant effect was recorded on copulation latency time between colonies at 3rd (ANOVA: F _(4, 10)_ = 2.05, P = 0.162) and 20th generation (ANOVA: F _(4, 10)_ = 0.466, P = 0.759) (Fig. 2b), as well as on mating duration at 3rd (ANOVA: F _(4, 10)_ = 0.537, P = 0.711) and 20th generation (ANOVA: F _(4, 10)_ = 1.334, P = 0.323) (Fig. 2c).

### Effects of “LEK-PC” treatment on the microbial community structure of medfly colonies

**Figure 3:**
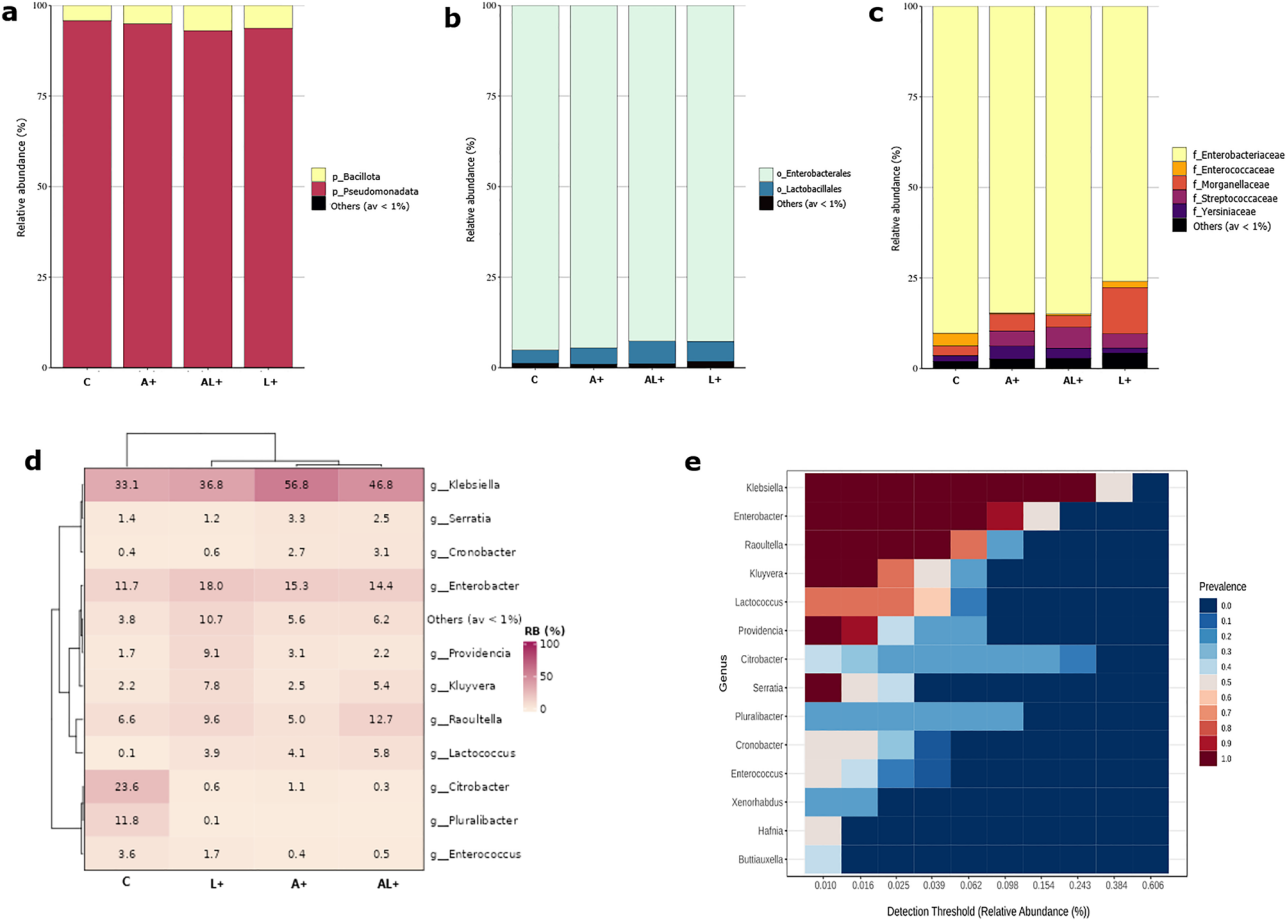
Transitions in the gut microbiota architecture of *C. capitata* following LEK-PC feeding. (**a**) Phylum, (**b**) order and (**c**) families relative abundance of the sterile male gut microbiota from LEK-PC feeding *C.capitata* colonies. (**d**) Heatmap showing the genera profile after LEK-PC feeding in different *C. capitata* colonies. (**e**) Core microbiome at the genera level of sterile male gut microbiota from LEK-PC feeding *C. capitata* colonies.

The gut bacterial communities of the four analyzed medfly colonies (A+, L+, AL+, and C) were classified in 4 phyla, 9 classes, 18 orders, 29 families and 55 genera. The metagenomic sequencing results revealed that the dominant bacteria at phylum, class, and order classification levels are consistent between all samples. At the phylum level, Pseudomonadata was the most dominant phylum in all simples (> 90%), followed by Bacillota, Actinobacteria, and a minor Bacteroides community (Fig. 3a, Supplementary Table S1). Similarly, at the class level, the major class in all samples was γ-Proteobacteria (> 90%) followed by Bacilli and a minor other community (Supplementary Table S1). At the order level, Enterobacterales predominated (> 90%) among medfly gut communities, followed by Lactobacillales and a minor other community (Fig. 3b; Supplementary Table S1). At the family level, *Enterobacteriaceae* was most dominant in all samples, followed by *Morganellaceae* in (A+) and (L+) (4.622% and 12.756% respectively), *Streptococcaceae* in (AL+) (5.76%) and *Enterococcaceae* in the (C) colony (3.56%) (Fig. 3c, Supplementary Table S1). At the genus classification level, divergences in the relative abundance of the OTUs were found accordingly to the “LEK-PC” feeding. Indeed, the long-term “LEK-PC” treatment significantly increased the relative abundance of *Enterobacter*, *Lactococcus*, *Klebsiella*, *Raoultella*, *Kluyvera*, *Hafnia,* and other minor community in (A+), (AL+) and (L+) medfly guts microbiota compared to the control (C) colony (Fig. 3d, Supplementary Table S1), instead, *Pluralibacter*, *Citrobacter,* and *Enterococcus* were significantly decreased. Although 55 genera were identified in the analyzed samples (Table S1), fourteen of them, Klebsiella, Enterobacter, Raoultella, Lactococcus, Providencia, Citrobecter, etc., constituted the core microbiota of the analyzed samples (Fig. 3e).

### Diversity of gut microbiota in “LEK-PC” treated *C. capitata* colonies

**Figure 4:**
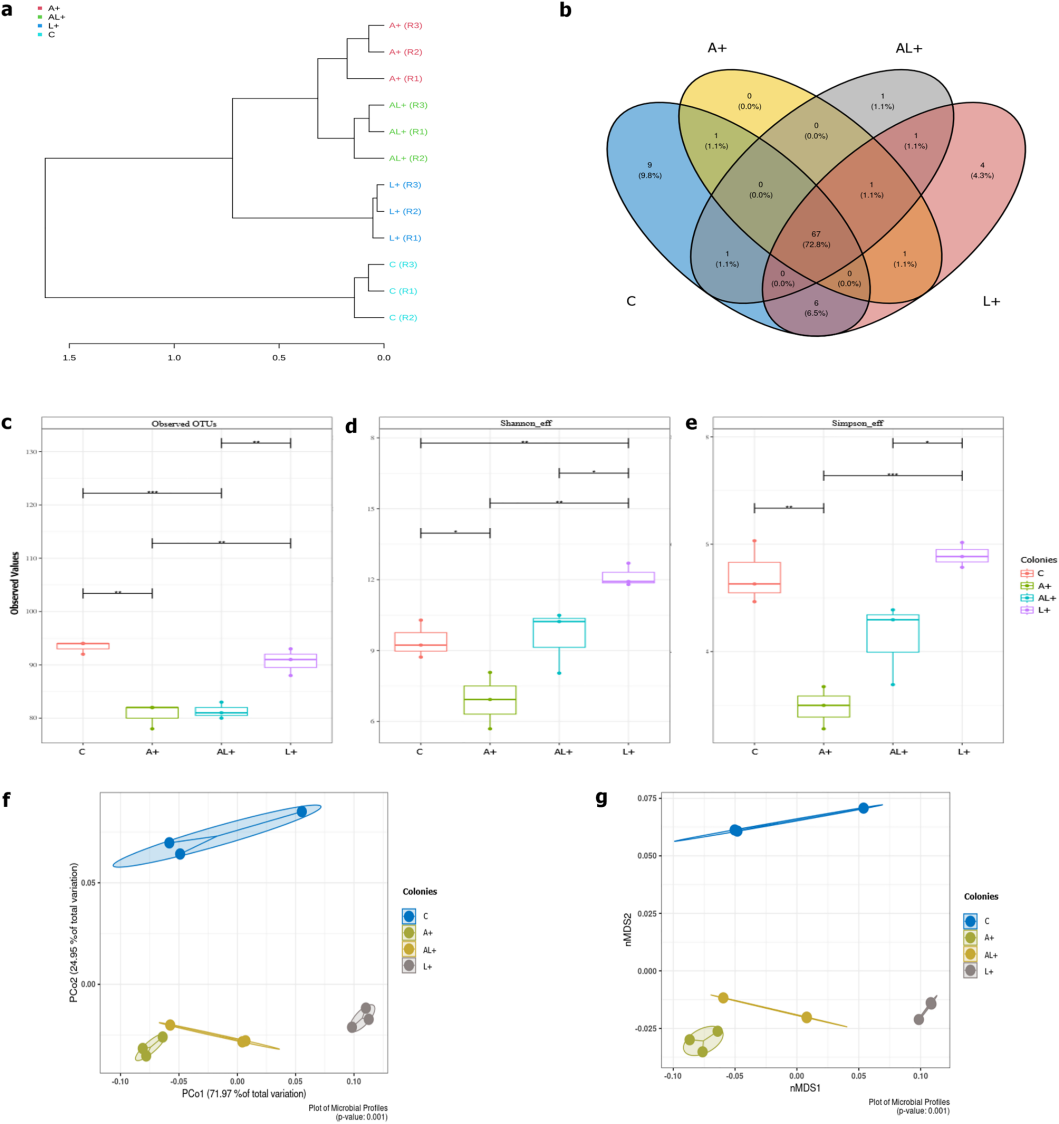
Effects of LEK-PC treatment on the intestinal microbiota diversity of *C.capitata* sterile males colonies. (**a**) Dendrogram representing hierarchical clustering distances based on Bray–Curtis dissimilarity indices calculated at genera level. (**b**) Venn diagram of sterile male gut bacterial genera from *C.capitata* LEK-PC feeding colonies. (**c**–**e**) Alpha diversity indices of sterile male gut bacterial communities from *C.capitata* LEK-PC feeding colonies. (**f**,**g**) Beta diversity indices of sterile male gut bacterial communities from *C.capitata* LEK-PC feeding colonies.

The dendogram (Fig. 4a) revealed the occurrence of two main clusters; cluster I included control colony samples, while cluster II contained all samples of LEK-PC feeding colonies (A+, L+ and AL+). From this result, it can be inferred that samples from LEK-PC feeding colonies exhibited similar abundances community at genera level, but substantially different from the C colony samples. According to the Venn diagram analysis, 67 genera of gut bacteria were shared between *C. capitata* colonies, (Fig. 4b).

The α-diversity analysis included the observed OTUs, Shannon effective, and Simpson effective indices, aiming to represent the richness and diversity of the microbiota community. Notably, the community richness and diversity in A+ and AL+ were lower than those in L+ and C, as indicated by the observed OTUs (Fig. 4c), Shannon effective index (Fig. 4d), and Simpson effective index (Fig. 4e). Indeed, both C and L+ colonies showed significant differences from A+ (P < 0.01, P < 0.001, respectively) and AL+ (P < 0.001, P < 0.01, respectively) in observed OTUs index (Fig. 4c). Regarding microbiota community diversity, samples from L+ exhibited significant differences compared to C (P < 0.01), A+ (P < 0.01), and AL+ (P < 0.05) in the Shannon effective index (Fig. 4d). In the case of the Simpson effective index, microbiota community diversity in L+ significantly differed from AL+ (P < 0.05) and A+ (P < 0.001) (Fig. 4e). It is noteworthy that a significant difference in α-diversity analysis was observed between C and A+ (P < 0.001), however, no significant difference was noted between A+ and AL+.

The principal coordinates analysis (PCoA) and non-metric multidimensional scaling (NMDS), which revealed the similarity measure of intestinal bacterial communities based on the phylogenetic distance, were performed baed on the generalized Unifrac distance matrixes (Fig). According the pairwise PERMANOVA analysis, PCoA (Fig. 4f) and NMDS (Fig. 4g) results revealed that *C.capitata* sterile males developed different bacterial profiles after “LEK-PC” treatment at different developmental stage (PERMANOVA; p < 0.001).

Linear discriminant analysis effect size (LEfSe) was used to screen out different taxa at various classification levels between each “LEK-PC” feeding group and the control based on a standard LDA (LDA > 2; p < 0.05). The analysis results showed that the gut microbiota composition between feeding groups (A+, AL+ and L+) and C had significant differences.

A total of 49 different abundant taxa were significantly enhanced in the L+ and C groups (Fig. S1a). Six families (*Bacillaceae, Streptococccaeae, Aeromonadaceae, Erwiniaceae, Hafniaceae* and *Morganellaceae*) and 20 genera (*Lactococcus, Enterobacter, Klebsiella, Morganella* and other) were relatively more abundant in the L+ group, and 9 families (*Enterococcus, Enterobacteriaceae, Pseudomonas* and other) and 14 genera (*Enterococcus, Citrobacter, Pluralibacter, Pseudomonas* and other) in the C group. However, 46 different abundant taxa were found to be amended in the A+ and C groups (Fig. S1b). There had been 15 distinguishable different families between the two groups, with significant abundance of *Streptococcaceae*, *Erwiniaceae*, *Hafniacea*, *Morganellaceae* and *Yerciniaseae* in A+, and *Enterococcacea*, *Lactobacillaceae*, *Enterobacteriaceae*, *Pectobacteriaceae* and other in C colony. Similarly, a total of 31 genera significantly enriched, *Lactococcus*, *Klebsiella*, *Erwinia*, *Morganella*, *Providencia* and other exhibited a relatively high abundance in A+ gut, and *Enterococcus*, *Citrobacter*, *Pluralibacter*, *Raoultella*, *Proteus* and other were relatively more abundant in C group. Finally, the comparison between AL+ and C revealed 51 different abundant taxa (Fig. S1c) with 6 significant abundance families (*Bacillaceae*, *Streptococcaceae*, *Erwiniaceae*, *Hafniaceae*, *Yersiniaceae* and *Morganellaceae*) in AL+ group and 12 different families in C group (*Enterococcus, Lactobacillaceae, Enterobacteriaceae, Pectobacteriaceae, Pseudomonadaceae* and other). In the genus classification level, a total of 33 genera were significantly enriched among them 15 genera (*Lactoccus, Klebsiella, Serratia, Morganella* and other) were relatively more abundant in AL+ feeding group and 18 genera in C group (*Pseudomonas*, *Enterococcus*, *Citrobacter*, *Pluralibacter* and other).

### Functional prediction analysis

To further understand the effect of long-term “LEK-PC” treatment across different life stages on the metabolic potential of medfly gut microbes, a metagenome prediction approach was used to determine probable functions of the bacterial communities through the MicFunPred web-based bioinformatics pipeline based on 16S rRNA sequencing data using KEGG level 1 to 3. The KEGG level 1 pathway indicated that the most functional prediction categories were related to metabolism (17.9 ± 0.18–18.32 ± 0.04%) followed by Genetic Information Processing (15.24 ± 0.02–15.86 ± 0.16%), Brite hierarchies (13.91 ± 0.01%–14.02 ± 0.01%), Environmental Information Processing (12.96 ± 0.03%–13.46 ± 0.14%), Cellular Processes (12.12 ± 0.26–13.05 ± 0.04%), Human Disease (11.46–11.73%) and Organismal Systems (8.83 ± 0.04%–8.96 ± 0.04%) in all four colonies (Supplementary Table S2). The KEGG level 2 pathway revealed that 30 of the 49 functional categories were differentially predicted across the four colonies (Supplementary Table S3). Among them, three pathways were significantly enhanced in A+ colony when compared to the control colony (membrane transport, environmental adaptation, and infectious diseases: virale), 15 pathways in AL+ colony when compared to the control colony (carbohydrate metabolism, amino acids metabolism, metabolism of cofactors, and vitamins, xenobiotics biodegradation, and metabolism, Nucleotide metabolism, and other), and two pathways were enriched in L+ colony when compared to the control colony (metabolism terpenoids and polyketides and unclassified: metabolism) (Supplementary Table S3). Whereas, the comparison between the “LEK-PC” feeding groups revealed no significant differences between A+ and L+, and between AL+ and L+. However, 10 functions in 14 KEGG pathways at level 2 differ significantly between AL+ and A+ (Supplementary Table S3). Moreover, in the KEGG level 3, the Dunn’s multiple comparison analysis showed that amino acids metabolism was the most enriched function in the AL+ colony compared to the C colony with 6 significantly improved functions, followed by the metabolism of carbohydrates with significantly 5 different functions, the metabolism of cofactors and vitamins, and the metabolism of other amino acids with 3 improved functions in AL+ colony compared to the control (Supplementary Table S4; Supplementary Fig. S2).

For additional investigation, we looked at the Carbohydrate active enzymes (CAZyme) analysis. The results revealed that there were 94 CAZyme including 55 glycoside hydrolases (GHs), of which 14 were significantly different between groups (p < 0.05), and the top five most abundant were GH1, GH2, GH3, GH4, and GH23 (Supplementary Table S5). In 24 glycosyl transferases (GTs), 14 were significantly different between the groups that were analyzed (p < 0.05), with GT2, GT4, and GT9 being the most abundant. Overall, there were significant differences between the groups for the other Cazymes that were examined, including four Carbohydrates Binding Modules (CBMs), four Carbohydrates Esterases (CEs), one enzyme with auxiliary activities (AAs), and four Polysaccharides Lyases (PLs) (p < 0.05).

## Discussion

The beneficial effect of feeding probiotics to improve the performance of medfly V8-GSS sterile males for SIT programs has been well demonstrated. Various bacteria that were isolated from the medfly intestinal tract were administered to insects through larval or adult diet either individually and/or in mixed cultures^12–17^. Although the effects of mass rearing and irradiation on the insect gut bacteriome have been previously described^8,11,16^, the effects on the medfly V8-GSS sterile male gut microbiota structure and population functionality during probiotic administration remain elusive. In order to assess the probiotic effect on insect performance and on the gut bacterial community using metagenomic sequencing, larval and/or adult dietary supplementation with a probiotic consortia named "LEK-PC" was investigated "in vivo" for 20 generations.

The supplementation of the selected probiotic bacteria *K. oxytoca*, *L. lactis*, and *Enterobacter* sp. in microbial consortia to larval and/or adult diets during 20 rearing generations improved the emergence, flight ability, survival under stress conditions, and mating competitiveness of medfly V8-GSS sterile males. This is in line with prior reports on the systemic beneficial effect of adding probiotics as food additives to improve medfly efficiency^12–17^. This improvement has been associated with participation of probiotics in the metabolism of the insect, in providing nutrients to improve insect development, and in restoring the various dysbiosis caused by mass rearing and irradiation on the commensal microbiome of the insect. Indeed, the addition of *Klebsiella oxytoca* to the medfly larval or adult diet for one generation improves the sterile mating competitiveness, mating latency, longevity under food and water starvation, flight ability, and the developmental duration of the immature stages^8,13,15,17^. Similarly, the adult or larval diet enrichment with *Enterobacter* sp. probiotic bacteria enhances various insect’s fitness parameters including pupal and adult productivity, developmental duration of the immature stages, pupal weight, flight ability, longevity under stress condition, and mating success^15–17^. These two bacteria were reported to be predominant commensal bacterial species in medfly gut microbiota, and substantially contribute to the insect’s nutrition via their pectinolytic activity to digest fruit sugar and diazotrophic capability to fix nitrogen^41^. When it comes to *Lactococcus lactis*, it is a lactic acid bacteria (LAB) that was previously isolated from the guts of the *C. capitata* wild population and chosen as a good potential probiotic strain by improving the QC parameters of medfly males^15,30^. The mechanisms underlying *L. lactis's* effects on insects' performance promotion, however, are still unknown.

In this study, we also examined the impact of the "LEK-PC" treatment at larval and/or adult stages on the intestinal gut microbiota diversity of V8-GSS sterile males after 20 generations of rearing. Despite the presence of LEK-PC medfly colonies, the results showed stable genera richness as well as notable variations in the membership diversity and community structure. Thus, a greater impact of the probiotic administration stage and diet on intestinal microbiota composition was observed in this study. These support earlier research indicating that insect feeding’s diet has a greater impact on shaping the gut microbiota structure of *C. capitata*^30,36^. Other factors, such as the developmental stage, age, geographic origin, medfly strain, rearing conditions, and irradiation process, contributed to the gut microbiota structuring of wild and laboratory medfly populations^8,11,30–32,36,44^. These factors were not taken into account for the current study because only adult male V8-GSS steriles from various "LEK-PC" feeding medfly colonies were used. Though the dominant phyla (Pseudomonadata), class (Gammaproteobacteria), Order (Enterobacteriales), Family (Enterobacteriaceae), and genus (*Klebsiella*) in the gut of medfly sterile males were highly consistent and stable between “LEK-PC” feeding colonies and the control, pairwise analysis revealed that probiotic administration at different developmental stages shifts the organization and abundance of the insect gut bacterial communities in different taxa classifications after 20 rearing generations. Previous studies describing the high dominance of Enterobacteriaceae family taxa in the intestinal bacterial community of *C. capitata* coincided with our results^8,34–36^. Additionally, the development and fitness of insects were linked to the presence of Enterobacteriaceae during various medfly developmental stages via vertical and horizontal transmission^27,29,35,48^. Nevertheless, in this study, there was an increase in the relative abundances of Streptococcaceae and Hafniacea, whereas the relative abundances of Enterobacteriaceae and Enterococcaceae exhibited varying decreases in colonies fed with probiotics compared to the control.. In the genus level, a high abundance of *Klebsiella* across all medfly colonies was shown. This genus, commonly reported as the most dominant symbiont in wild population of *C. capitata*^11,32–34^, has a pivotal role in providing the host with nitrogen resources and the maintenance of gut homeostasis by preventing the establishment of pathogenic bacteria^26,29,40,44^. These positive features related to *Klebsiella* in the gut allowed its exploitation as a probiotic to restore the biological quality parameters of medfly sterile males and other insects that had been affected by mass rearing and irradiation in SIT programs^8,9^. Moreover, *Enterobacter* abundance in probiotic feeding and control colonies agrees with the high relative abundance reported of this genus in the wild and laboratory *C. capitata* strain^15,16,29–38^. Mainly represented by *E. cloacae*, *E. agglomerans* and *E. aeorgenes*, *Enterobacter* species maintain beneficial relationships with fruit flies according to their abilities to hydrolyze complex carbohydrates, catalyze nitrogen fixation, produce vitamins and pheromones, and provide protective functions to their host^1,49,50^. In contrast, the relative abundance of *Pluralibacter* and *Citrobacter* decreased following probiotic administration compared to the control. Nonetheless, it is noteworthy that these genera, in collaboration with other members of the *Enterobacteriacea* were identified to be stable endosymbiotic species for medfly involved in essential nutrient synthesis, nitrogen fixation, and recycling of nitrogenous waste products into useful compounds^41,49^. In addition to members of the *Enterobacteriacea* family, the *Enterococcus* genus (*Enterococcacea*) had a lower abundance in probiotic feeding colonies compared to the control. This genus has previously been detected in the guts of laboratory Vienna 8 Faster Development-GSS (V8 FD-GSS) and wild medfly populations^11,34^ as well as in many other insects. It protects against pathogens and toxic compounds enhancing host fitness ^51–53^.

It is well known that the insect gut microbiota governs many physiological functions contributing to the host’s development, pathogen resistance, nutrition, and physiology^1^. Based on metagenomic sequencing data and functional classification databases, we used MicFunPred to predict the potential changes in functions of the gut microbiota reorganization in response to various manifestations of dysbiosis associated to probiotic administration stage, that are typically associated with different taxa. In this study, the main metabolic pathways found in all medfly colonies are consistent with the reported nutritional role played by medfly-associated symbionts^27,28,41,49,54^. As expected, pairwise comparisons between the different colonies revealed that the AL+ colony exhibited the highest number of improved metabolic pathways compared to the control, in particular in carbohydrates, amino acids, cofactors, and vitamin metabolism. As previously reported, both *Enterobacter* sp. and *Klebsiella oxytoca* belong to the *Enterobacteriacea* family members and play an important role in carbohydrate and protein digestion^27,28,41^. *Lactococcus* has also been shown to contribute to insect nutrition due to its high performance in degrading pectin-rich fruits and a variety of sugars^30,55^. Consequently, the association of those three species in probiotic consortium may provide various metabolic benefits to the medfly but further research is needed to identify these functions.

In this study, we also identified a series of carbohydrates-active enzymes in different probiotic feeding colonies. We found that GHs were the most abundant enzymes expressed in all samples. These findings coincide with those of Silva et al.^56^ who associate enzymatic activities to digest carbohydrates in *C. capitata* larvae and adults to specific glycosil-hydrolases. Moreover, divers GHs enzymes including β-galactosidase (GH2), β-glucosidase (GH3), α-amylase (GH13_10), α-glucosidase (GH4), α-mannosidase (GH38) etc., were significantly more abundant in AL+ colony compared to the control. In coincidence with Estes et al.^57^ findings, *Enterobacter* sp., isolated from wild male olive flies (*Bactrocera olea*), has a complete set of enzymes for degrading a variety of sugars in its genome which would allow it to be a strain with a high probiotic potential for SIT programs. As a result, it can be evidenced that the simultaneous LEK-PC administration to larvae and adults of V8-GSS *C. capitata* strain during 20 generations of rearing is optimal for probiotic successful gut-colonization and positive modulation of the insect microbiome through activation of beneficial metabolic pathway and carbohydrates-active enzymes expression.

## Conclusion

In conclusion, the present study evidenced that the LEK-PC treatment improves the fitness parameters of *C. capitata* over generations regardless the stage of administration. Moreover, the 16S rRNA metagenomics data demonstrated that the intestinal microbiota structure was significantly influenced by the probiotic treatment while still maintaining a stable core dominant community of *Enterobacteriacea*. Last but not least, the AL+ colony had the most improved potential functions in terms of gut microbes as well as the carbohydrates active enzymes most improved potential functions. Our research opens up new avenues of investigation into the potential of probiotics to improve sterile insect gut microbiota structure and function in SIT programs.

## Materials and methods

### Origin and preparation of the “L.E.K” probiotic consortium

The bacterial strains, *Lactococcus lactis* (KY807048), *Klebsiella oxytoca* (KY810531), and *Enterobacter* sp. (KY810513) were previously isolated from *Ceratitis capitata* adult guts and selected as the best potential probiotic strains according to their “in vitro” and “in vivo” probiotic properties^15^. From their respective glycerol stock, each bacteria was streaked onto a separate Luria Bertani (LB) agar plate and incubated overnight at 37 °C. A single colony from each plate was seeded in separate tubes containing LB broth which was then incubated overnight at 37 °C, and stored at 4 °C. For the probiotic blend preparation, each of the bacterial species was grown individually by adding 1 ml from its respective bacterial culture solution to 100 ml of sterile LB broth (1% (v/v) inoculum). The three bacterial cultures were kept at 37 °C to allow the bacteria to grow in numbers up to the mid-log phase. To determine the approximate number of bacterial cells (in terms of colony-forming units, CFU), a 1 ml sample was removed from the solution and was tenfold serially diluted. The dilutions were spread on LB agar and incubated for 24 h at 37 °C for counting (to reach final concentrations of 10^9^ CFU/ml). The remainder of each solution was washed free of medium and re-suspended in sterile distilled water. The “L.E.K” probiotic consortium (LEK-PC) was set by mixing the three bacterial cultures for a final concentration of 10^9^ CFU/ml each.

### Establishment of medfly colonies and generation maintenance

The experiment was carried out using V8-GSS medfly strain which was maintained in the Tunisian medfly rearing facility. The LEK-PC treatments cohorts of V8-GSS in this study were designed in 4 colonies: (C) the control colony reared on LEK-PC free larval and adult diets; (L+) the larval LEK-PC enriched diet colony reared according to the following steps: V8-GSS eggs were seeded on Tanaka larval diet (28% wheat bran, 14% sugar, 7% Torula yeast, 1% HCl 37%, 0.2% sodium benzoate, and 50% water) containing 10^9^ CFU/g of LEK-PC for each generation. Emerged (L+) adults were transferred to a polystyrene cage with a mesh window and fed with a mixture of sugar and hydrolyzed yeast extract (3:1); (A+) the adult LEK-PC enriched diet colony reared according to the following steps: V8-GSS adults were fed with autoclaved liquid adult diet (sugar, hydrolyzed yeast and water (3:1:40)) containing 10^9^ CFU/ml of LEK-PC for each generation from F1 to F20. The female (A+) laid eggs were collected and seeded on a standard Tanaka larval diet, and (AL+) both adults and larvae are reared on a LEK-PC enriched diet for each generation. All colonies were reared over 20 generations. The environmental conditions for larval development were 23 °C and 80% RH, and 25 °C and 65% RH for pupae and adults.. Sterile males were obtained by exposing the collected pupae from each colony to an irradiation dose of 110 Gy, 2 days before emergence. The schematic generational maintenance of probiotic-feeding medfly colonies is shown in Fig. 1. The wildish type *C.capitata* strain (Cw) used in this study was obtained from infested citrus fruits collected from the Cap Bon region of (Tunisia)^15^. This strain has been reared for approximately 10 generations at the Tunisian Medfly rearing facility located within the National Center for Nuclear Science and Technology (CNSTN).

### Gut dissection, DNA isolation, and illumina Miseq sequencing

Five-day-old sterile males from each colony's F20 generation were surface sterilized and their guts were extracted sterilely using a stereomicroscope. Fifteen guts were pooled (from crop to hindgut without Malpighian tubes) to create one sample (replicate). There were three replicates per treatment. Total DNA extraction was performed using a DNA kit (BioBasic) according to the manufacturer’s instructions. The quality and concentration of the extracted DNA were analyzed using a NanoDrop 2000 Spectrophotometer at 260 nm. To assess the impact of LEK-PC treatment on sterile male intestinal microbiome profiling at the different developmental stages of V8-GSS medfly after 20 generations, bacterial 16S ribosomal DNA amplicons were prepared by amplification of the 16S rRNA gene V4 variable region with PCR primers U515F (GTGCCAGCMGCCGCGGTAA) and 805R (GGACTACHVGGGTWTCTAAT)^58^ using the HotStarTaq Plus Master Mix Kit (Qiagen, USA) under the following conditions: 94 °C for 3 min, followed by 30–35 cycles of 94 °C for 30 s, 53 °C for 40 s and 72 °C for 1 min, after which a final elongation step at 72 °C for 5 min was performed. Following amplification, PCR products were examined in 2% agarose gel to determine the success of amplification and the relative band intensity. Multiple samples are pooled together in equal proportions based on their molecular weight and DNA concentrations. Pooled samples were purified using calibrated Ampure XP beads. The pooled and purified PCR product was used to prepare illumina DNA library. Sequencing was performed at MR DNA (http://www.mrdnalab.com, Shallowater, TX, USA) on a MiSeq following the manufacturer’s guidelines. Sequence data were processed using MR DNA analysis pipeline.

### Investigation of LEK-PC impact on medfly quality control (QC) parameters

The biological quality of the medfly colonies was assessed. All assays were carried out on the 3rd earlier generation (F3) and the 20th later generation (F20) in accordance with the procedures outlined in the Product Quality Control for Sterile Mass-Reared and Released Tephritid Fruit Flies^59^.

### Pupal weight

Groups of 100 pupae were collected from each colony and weighted 2 days before emergence. Three replicates were set up per colony.

### Flight ability

To determine the effect of the LEK-PC treatments on the emergence and flight capacity, 100 irradiated pupae from each colony were placed in a black plexiglass cylinder. Emerging flies were collected continuously while the cylinders were placed in plexiglass cages 30 cm wide by 40 cm deep. Non-emerged and partially emerged adults, adults with malformations, non-fliers, dead flies, and flies that were able to leave the cylinder while flying were all recorded during the 72-h test. This test was replicated thrice with the environmental conditions maintained at 25 °C, 65% RH, and a light intensity of 1500 lx.

### Survival under stress

Samples of 50 sterile males from each colony were placed in petri dishes with a 15 mm opening covered with netting within 2 h of emergence, with no food or water. The dishes were maintained in the dark at 25 °C and 65% RH. Dead and live flies were counted after 48 h. Three replicates were made for each group.

### Female fecundity

Three days after adult emergence, 50 males and 50 females from each colony were placed in a plastic egging cage (15 cm × 15 cm × 15 cm) with a sugar and yeast hydrolysate mixture of 3:1 (wt:wt), respectively. Water was added through a soaked sponge. For 5 days, produced eggs were collected and counted daily from each cage. Egg productivity per female per day was estimated by dividing the number of produced eggs by the number of females per day for each egging cage. A combined sample of 300 eggs was collected over a period of 5 days from each colony to assess egg hatch. Three replicates, each consisting of an egging cage, were conducted for each colony.

### Mating competitiveness

The mating test was performed between V 8-GSS sterile males collected from each LEK-PC treatment (A+, AL+ and L+), and control (C), against wildish males (Cw) for the mating of wildish females. For this purpose, 24 h after eclosion, the V8-GSS sterile males from each colony, the wildish males, and females were separated by sex and provided with water and a mixture of sucrose and yeast powder (3:1) until sexual maturity (11–12 days for wildish flies and 5–8 days for V 8-GSS flies). To distinguish between each group, wildish and V 8-GSS males were marked with different nontoxic dyes on the thorax one day before testing. The mating test was performed by releasing 25 males of each of the four colonies (25 wildish males and 25 sterile males from each treated colony) into a Plexiglas cage (30 × 30 × 30 cm) followed by the introduction of 25 virgin wildish females 5–10 min later. The test began at 9:00 a.m. and was visually inspected each 15 min for 4 h. Mating pairs were collected into transparent plastic vials. The percentage of copulatory success was estimated by counting the number of matings achieved by the probiotic-enriched males when competing for wildish virgin females against the wildish male for each group. This test was replicated thrice for each probiotic male colony.

### Bioinformatic and statistical analyses

Sequence data were processed using the MR DNA analysis pipeline (MR DNA, Shallowater, TX, USA). In summary, sequences were joined, sequences < 150 bp removed, and sequences with ambiguous base calls removed. Sequences were quality filtered using a maximum expected error threshold of 1.0 and dereplicated. The dereplicated or unique sequences were denoised; unique sequences identified with sequencing or PCR point errors are removed, followed by chimera removal, thereby providing a denoised sequence or zOTU. Final zOTUs were taxonomically classified using BLASTn against MR DNA’s customized, proprietary database derived from the 2016 version of RDPII (http://rdp.cme.msu.edu) and NCBI (http://www.ncbi.nlm.nih.gov).

Alpha diversity was estimated through Observed OTUs, Shannon effective, and Simpson effective indices. Pairwise ANOVA was used to identify significant differences of α-diversity indices between the different groups. Beta diversity was analyzed to investigate the microbial community structural variation using Principal coordinate analysis (PCoA) and non-metric multidimensional scaling (NMDS) based on Generalized Unifrac distances metric. Statistically significant differences between samples were assessed with permutational multivariate analysis of variance (PERMANOVA) using 999 permutations. Bar charts, alpha and beta diversity, heatmaps, core microbiome, dendrogram and Venn diagram were calculated and plots generated using the web-based tool Microbiome Analyst (https://www.microbiomeanalyst.ca) and qplots pipeline (http://www.qplots.eu). The bacterial genera identified and the linkage between bacterial taxa and predicted functions are shown in a figure drawn by Circos (http://mkweb.bcgsc.ca/tableviewer/). Linear discriminant analysis effect size (LEfSe, LDA > 2) was done for comparison of microbial variations using online tool (https://huttenhower.sph.harvard.edu/galaxy). The significance of differences of gut microbiota structures and functional profiles among groups based on the relative abundance were assessed by one-way ANOVA and Tukey–Kramer post hoc test (for normally distributed data) and Kruskal–wallis test and Dunn’s multiple comparison test (for no-normally distributed data).

The prediction functional genomic analysis of the microbial community present in each medfly colony gut was carried out by using the MicFunPred web-based bioinformatic pipeline available at (http://micfunpred.microdm.net.in/)^60^. The Kyoto Encyclopedia of Genes and Genomes (KEGG) database was used for functional classification. The annotation information for carbohydrates active enzymes (CAZymes) was obtained from the CAZy database^61^.

Quality control parameters including pupal weight, emergence, flight ability, survival under stress conditions, egg production and hatching percentage, mating duration, mating latency and mating percentage data were normally distributed prior to analysis. One-way ANOVA followed by Sidak’s post hoc pairwise comparisons was performed to identify significant differences between the colonies in each rearing generation (F3 and F20). Student *t*-test was performed to identify significant differences between the two rearing generations (F3 and F20) for each colony.

### Supplementary Information

Supplementary Figure S1.

Supplementary Figure S2.

Supplementary Tables.

Supplementary Table S5.

Supplementary Legends.

## Data Availability

All data generated or analyzed during this study are included in this article and its Supplementary Information files. Raw 16S rRNA reads were deposited in the NCBI Sequence Read Archive (SRA) database (Submission ID: SUB13774114; BioProject ID: PRJNA1006652).
