# Supplementary figures and images for: Probiotic consortium modulating the gut microbiota composition and function of sterile Mediterranean fruit flies
Source: Sci Rep. 2024 Jan 11;14:1058. doi: 10.1038/s41598-023-50679-z (PMC10784543; doi:10.1038/s41598-023-50679-z)

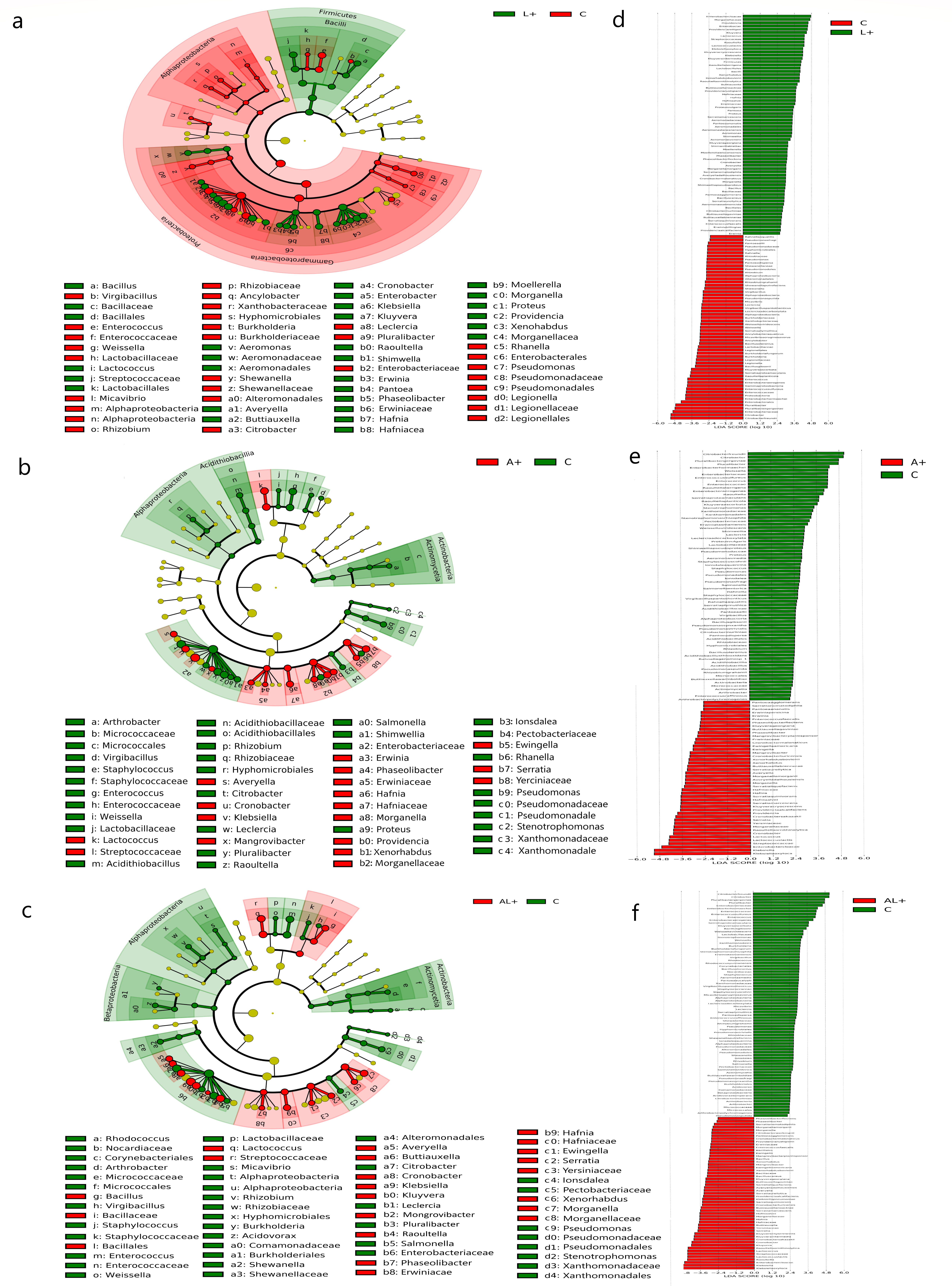

Supplement: Supplementary file 1 — Supplementary Figure S1. [file 41598_2023_50679_MOESM1_ESM.tiff]

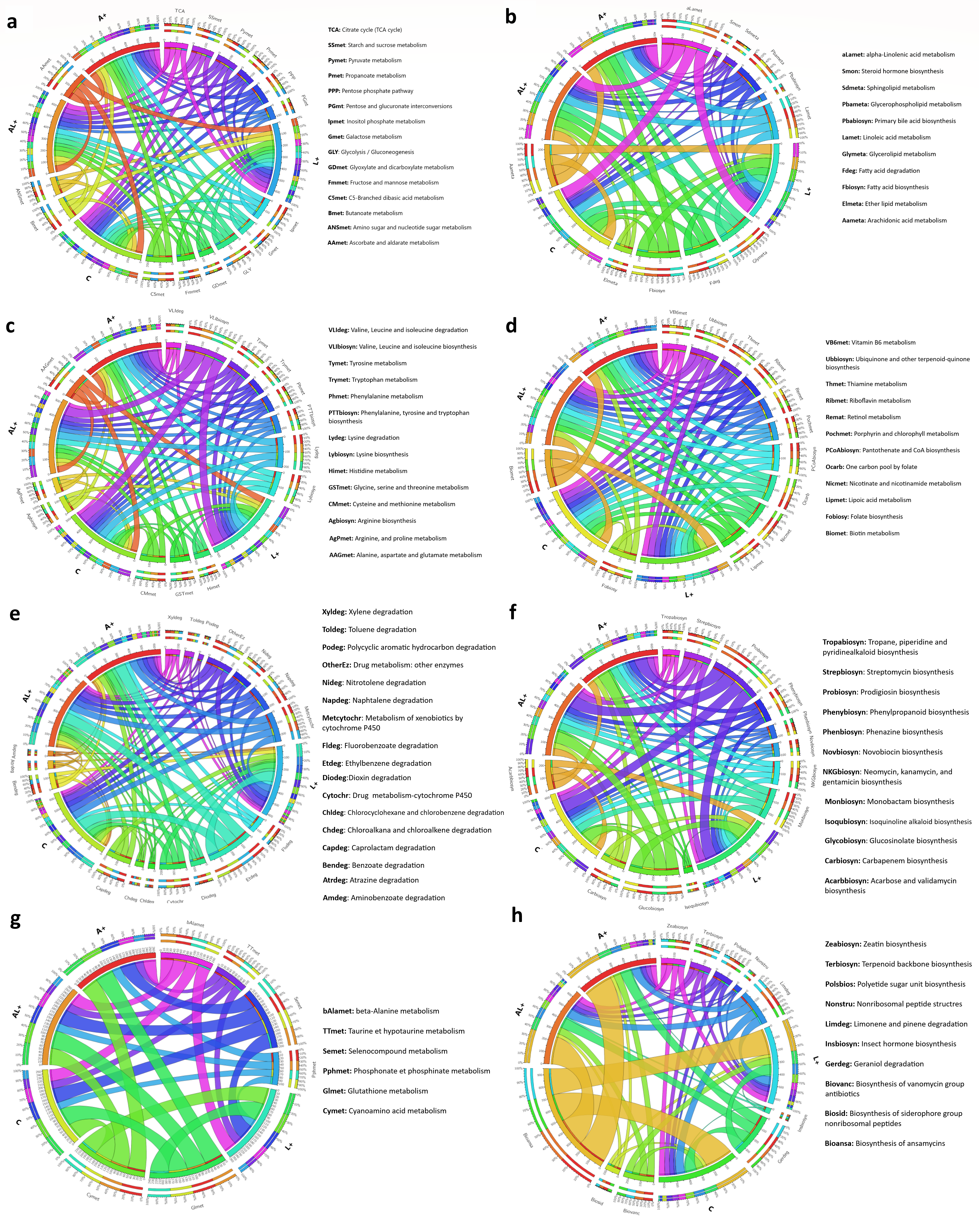

Supplement: Supplementary file 2 — Supplementary Figure S2. [file 41598_2023_50679_MOESM2_ESM.tiff]
