# Supplementary material for: Probiotic consortium modulating the gut microbiota composition and function of sterile Mediterranean fruit flies
Source: Sci Rep. 2024 Jan 11;14:1058. doi: 10.1038/s41598-023-50679-z (PMC10784543; doi:10.1038/s41598-023-50679-z)
Supplement: Supplementary file 4 — Supplementary Table S5. [file 41598_2023_50679_MOESM4_ESM.docx]

Table S5: Abundance of Carbohydrate active enzymes in guts microbes of different *C. capitata* colonies

| **CAZy family** | | **Abundance** | | | | **Pair-wise comparisons** | **Known activities (CAZy EC number)** |
| --- | --- | --- | --- | --- | --- | --- | --- |
|  |  | **A+** | **AL+** | **L+** | **C** |  |  |
| **Glycoside Hydrolase (GHs)** | GH0 | 353,33 ± 7,33 | 338,33 ± 9,52 | 385 ± 7,21 | 392,33 ± 46,44 | ns | Glycoside hydrolases not yet assigned to a family. |
|  | GH1 | 5440,66 ± 166,86 | 7034,66 ± 193,09 | 7586,66 ± 306,9 | 4908 ± 416,5 | ns | β-glucosidase (EC 3.2.1.21) ; β-galactosidase (EC 3.2.1.23) ; β-mannosidase (EC 3.2.1.25) ; β-glucuronidase (EC 3.2.1.31) ; β-xylosidase (EC 3.2.1.37) ; and others. |
|  | GH2 | 2467 ± 63,57 | 3627,66 ± 104,67 | 4039,33 ± 205,45 | 7418,66 ± 532,01 | AL+ *vs* C (p-value : 0.0134) | β-galactosidase (EC 3.2.1.23) ; β-mannosidase (EC 3.2.1.25) ; β-glucuronidase (EC 3.2.1.31) ; α-L-arabinofuranosidase (EC 3.2.1.55) ; and others. |
|  | GH3 | 3405,33 ± 121,77 | 3709,66 ± 97,4 | 4315 ± 40,5 | 3152,33 ± 149,78 | AL+ *vs* C (p-value : 0.0279) | β-glucosidase (EC 3.2.1.21) ; xylan 1,4-β-xylosidase (EC 3.2.1.37) ; β-glucosylceramidase (EC 3.2.1.45) ; β-N-acetylhexosaminidase (EC 3.2.1.52) ; and others. |
|  | GH4 | 3459,66 ± 137,17 | 3421 ± 103,02 | 4172,33 ± 48,96 | 3047,66 ± 143,39 | AL+ *vs* C (p-value : 0.0194) | Maltose-6-phosphate glucosidase (EC 3.2.1.122); α-glucosidase (EC 3.2.1.20); α-galactosidase (EC 3.2.1.22); 6-phospho-β-glucosidase (EC 3.2.1.86); and others. |
|  | GH5_13 | 412,66 ± 8,21 | 275,66 ± 10,74 | 94,66 ± 8,64 | 454,33 ± 53,25 | L+ *vs* C (p-value : 0.0395) | β-D-galactofuranosidase (EC 3.2.1.146); α-L-arabinofuranosidase (EC 3.2.1.55) |
|  | GH8 | 661 ± 29,19 | 974,33 ± 30,36 | 412,33 ± 7,85 | 1081 ± 154,42 | ns | chitosanase (EC 3.2.1.132); cellulase (EC 3.2.1.4); licheninase (EC 3.2.1.73); endo-1,4-β-xylanase (EC 3.2.1.8); reducing-end-xylose releasing exo-oligoxylanase (EC 3.2.1.156) |
|  | GH13_5 | 1070±91,24 | 1629,66±47,57 | 1127±18,33 | 4383±562,53 | A+ *vs* C (p-value : 0.0395) | [retaining] maltopentaose-producing α-amylase (EC 3.2.1.-); [retaining] α-amylase (EC 3.2.1.1); [retaining] maltotriose-producing α-amylase (EC 3.2.1.116); and others. |
|  | GH13_9 | 1165,66 ± 97,86 | 1745,66 ± 55,12 | 1264,33 ± 21,42 | 4800,33 ± 615,64 | A+ *vs* C (p-value : 0.0395) | [retaining] α-1,4-glucan branching enzyme (EC 2.4.1.18) |
|  | GH13_10 | 410 ± 9,45 | 294 ± 14,97 | 140,33 ± 4,7 | 484,66 ± 56,13 | AL+ *vs* C (p-value : 0.0279) | α-amylase (EC 3.2.1.1) ; [retaining] maltooligosyltrehalose trehalohydrolase (EC 3.2.1.141) |
|  | GH13_11 | 1714,33 ± 107,92 | 2212 ± 57,02 | 1412 ± 16,5 | 5306 ± 658,82 | L+ *vs* C (p-value : 0.0134) | 4-α-glucanotransferase / amylomaltase (EC 2.4.1.25) ; α-glucosidase (EC 3.2.1.20) ; isoamylase (EC 3.2.1.68) |
|  | GH13_19 | 1067,66 ± 95,08 | 1688 ± 51,53 | 1177,66 ± 20,38 | 4548 ± 586,5 | A+ *vs* C (p-value : 0.0395) | [retaining] maltopentaose-producing α-amylase (EC 3.2.1.-);[retaining] α-amylase (EC 3.2.1.1); [retaining] maltotetraose-producing α-amylase (EC 3.2.1.60); and others. |
|  | GH13_21 | 616 ± 82,86 | 1326,66 ± 54,77 | 1024,33 ± 24,74 | 3883,66 ± 520,57 | A+ *vs* C (p-value : 0.0134) | [retaining] α-amylase (EC 3.2.1.1); α-glucosidase (EC 3.2.1.20) |
|  | GH13_26 | 411,66 ± 11,6 | 295,66 ± 17,14 | 143 ± 5,19 | 486,33 ± 54,84 | L+ *vs* C (p-value : 0.0194) | malto-oligosyltrehalose synthase (EC 5.4.99.15) |
|  | GH13_29 | 1160,66 ± 46,04 | 1236,33 ± 26,39 | 979,66 ± 9,93 | 3776,66 ± 481,49 | L+ *vs* C (p-value : 0.0134) | α-glucosidase (EC 3.2.1.20); [retaining] a,α-trehalose-6-phosphate hydrolase (EC 3.2.1.93) |
|  | GH13_18 | 826 ± 16,62 | 551 ± 21,45 | 187,33 ± 17,29 | 907,66 ± 105,38 | L+ *vs* C (p-value : 0.0395) | [retaining] α-glucoside phosphorylase (EC 2.4.1.-) ; [retaining] sucrose 6(F)-phosphate phosphorylase (EC 2.4.1.329) ; glucosylglycerate phosphorylase (EC 2.4.1.352); and others. |
|  | GH18 | 1650,66 ± 66.7 | 1261,66 ± 39,76 | 667 ± 49,64 | 1050,33 ± 112,09 | A+ *vs* L+ (p-value : 0.0134) | chitinase (EC 3.2.1.14) ; lysozyme (EC 3.2.1.17) ; endo-β-N-acetylglucosaminidase (EC 3.2.1.96) ; and others. |
|  | GH19 | 700,66 ± 31,46 | 1020,33 ± 36,95 | 467,66 ± 6,56 | 1156,66 ± 162,59 | ns | chitinase (EC 3.2.1.14) ; lysozyme (EC 3.2.1.17) |
|  | GH102 | 2184,66 ± 102,15 | 2418,33 ± 64,7 | 3381 ± 32,95 | 4600 ± 510,58 | A+ *vs* C (p-value : 0.0194) | peptidoglycan lytic transglycosylase (EC 3.2.1.-) |
|  | GH103 | 2383 ± 103,9 | 2570,33 ± 73,21 | 3499 ± 35,9 | 4858 ± 535,21 | A+ *vs* C (p-value : 0.0194) | peptidoglycan lytic transglycosylase (EC 3.2.1.-) |
|  | GH105 | 1099,33 ± 94,62 | 1880 ± 59,35 | 1232 ± 23,51 | 4467 ± 578,01 | A+ *vs* C (p-value : 0.0395) | unsaturated rhamnogalacturonyl hydrolase (EC 3.2.1.172); d-4,5-unsaturated β-glucuronyl hydrolase (EC 3.2.1.-); d-4,5-unsaturated α-galacturonidase (EC 3.2.1.-) |
|  | GH108 | 351,66 ± 7,21 | 236,66 ± 9,24 | 80,66 ± 6,56 | 390 ± 46,13 | L+ *vs* C (p-value : 0.0395) | N-acetylmuramidase (EC 3.2.1.17) |
|  | GH77 | 1110,33 ± 88,41 | 1656,33 ± 49,8 | 1159,33 ± 19,34 | 4345,33 ± 554,82 | A+ *vs* C (p-value : 0.0395) | amylomaltase or 4-α-glucanotransferase (EC 2.4.1.25) |
|  | GH94 | 352 ± 8,08 | 236,33 ± 9,52 | 81,33 ± 6,76 | 389 ± 45,56 | L+ *vs* C (p-value : 0.0395) | cellobiose phosphorylase (EC 2.4.1.20) ; laminaribiose phosphorylase (EC 2.4.1.31) ; cellodextrin phosphorylase (EC 2.4.1.49) ; and others. |
|  | GH24 | 4587,33 ± 181,1 | 5910,33 ± 167,58 | 7569,66 ± 278,35 | 10070,33 ± 890,27 | A+ *vs* C (p-value : 0.0134) | lysozyme (EC 3.2.1.17) |
|  | GH23 | 59118,66 ± 5359,02 | 55365 ± 3940,7 | 57014 ± 1263,63 | 58013,66 ± 3315,65 | ns | lysozyme type G (EC 3.2.1.17); peptidoglycan lyase (EC 4.2.2.n1) also known in the literature as peptidoglycan lytic transglycosylase; chitinase (EC 3.2.1.14) |
|  | GH13_20 | 102,33 ± 36,04 | 116,33 ± 13,93 | 413 ± 59,91 | 820,66 ± 135,54 | A+ *vs* C (p-value : 0.0395) | [retaining] cyclic α-1,6-maltosyl-maltose hydrolase (EC 3.2.1.-);[retaining] reducing-end specific α-glucosidase (EC 3.2.1.-);[retaining] α-amylase (EC 3.2.1.1); and others. |
|  | GH13_31 | 959,33 ± 82,26 | 761,33 ± 17,13 | 1199,33 ± 163,68 | 2837,33 ± 299,53 | AL+ *vs* C (p-value : 0.0134) | oligosaccharide α-4-glucosyltransferase (EC 2.4.1.161) ; palatinase (EC 3.2.1.-) ; [retaining] α-amylase (EC 3.2.1.1) ; [retaining] oligo-α-1,6-glucosidase (EC 3.2.1.10) ; α-glucosidase (EC 3.2.1.20) ; β-glucosidase (EC 3.2.1.21) ; and others. |
|  | GH170 | 122,33 ± 43,41 | 139 ± 16,82 | 495,66 ± 70,63 | 984 ± 162,35 | A+ *vs* C (p-value : 0.0395) | 6-phospho-N-acetylmuramidase (EC 3.2.1.-) |
|  | GH154 | 661,33 ± 28,6 | 973,33 ± 30,05 | 411,66 ± 8,25 | 1079,33 ± 154,32 | ns | β-glucuronidase (3.2.1.31) |
|  | GH42 | 1028 ± 15,01 | 1007 ± 15,56 | 1012,33 ± 7,44 | 1079 ± 153,44 | ns | β-galactosidase (EC 3.2.1.23); α-L-arabinopyranosidase (EC 3.2.1.-) |
|  | GH109 | 16,66 ± 1,45 | 7 ± 1 | 8,66 ± 0,33 | 15,33 ± 2,02 | ns | α-N-acetylgalactosaminidase (EC 3.2.1.49) |
|  | GH125 | 91,66 ± 32,37 | 104,33 ± 12,41 | 369 ± 53,66 | 731,66 ± 120,76 | A+ *vs* C (p-value : 0.0395) | exo-α-1,6-mannosidase (EC 3.2.1.-) |
|  | GH127 | 660,66 ± 28,89 | 973 ± 29,77 | 411,33 ± 8 | 1078,66 ± 154,09 | ns | β-L-arabinofuranosidase (EC 3.2.1.185); 3-C-carboxy-5-deoxy-L-xylose (aceric acid) hydrolase (EC 3.2.1.-) |
|  | GH13 | 352,66 ± 7,17 | 370,66 ± 24,65 | 415,66 ± 25,72 | 390 ± 45,39 | ns | α-amylase (EC 3.2.1.1) ; pullulanase (EC 3.2.1.41) ; cyclomaltodextrin glucanotransferase (EC 2.4.1.19) ; trehalose-6-phosphate hydrolase (EC 3.2.1.93); and others. |
|  | GH130 | 351,66 ± 6,69 | 235 ± 8,96 | 81 ± 7,76 | 387 ± 45,56 | L+ *vs* C (p-value : 0.0395) | β-1,4-mannosylglucose phosphorylase (EC 2.4.1.281) ; β-1,4-mannooligosaccharide phosphorylase (EC 2.4.1.319) ; β-1,4-mannosyl-N-acetyl-glucosamine phosphorylase (EC 2.4.1.320); and others. |
|  | GH13_16 | 40 ± 2,08 | 47 ± 6,11 | 58 ± 3,21 | 77,66 ± 9,2 | A+ *vs* C (p-value : 0.0322) | trehalose synthase / maltose glucosylmutase (EC 5.4.99.16) |
|  | GH13_3 | 37,66 ± 2,4 | 43,66 ± 5,78 | 54 ± 3,21 | 72,66 ± 8,17 | A+ *vs* C (p-value : 0.0389) | α-1,4-glucan : phosphate α-maltosyltransferase (EC 2.4.99.16) |
|  | GH144 | 351,66 ± 6,69 | 235 ± 8,96 | 81,33 ± 7,85 | 388 ± 45,56 | L+ *vs* C (p-value : 0.0395) | Endo-β-1, 2-glucanase (EC 3.2.1.71); β-1, 2-glucooligosaccharide sophorohydrolase (EC 3.2.1.-) |
|  | GH20 | 417 ± 36,513 | 349,66 ± 9,2 | 189,66 ± 16,38 | 81,66 ± 5,17 | A+ *vs* C (p-value : 0.0194) | β-hexosaminidase (EC 3.2.1.52); lacto-N-biosidase (EC 3.2.1.140); β-1,6-N-acetylglucosaminidase (EC 3.2.1.-); β-6-SO3-N-acetylglucosaminidase (EC 3.2.1.-) |
|  | GH28 | 381,33 ± 27,48 | 909,66 ± 34,27 | 408,33 ± 13,01 | 853,33 ± 134,43 | ns | polygalacturonase (EC 3.2.1.15); α-L-rhamnosidase (EC 3.2.1.40); exo-polygalacturonase (EC 3.2.1.67); exo-polygalacturonosidase (EC 3.2.1.82); rhamnogalacturonase (EC 3.2.1.171); and others. |
|  | GH31 | 5028,33 ± 92,33 | 5602,66 ± 60,49 | 5667,66 ± 588,9 | 4878,33 ± 423,83 | ns | α-glucosidase (EC 3.2.1.20); α-galactosidase (EC 3.2.1.22); α -mannosidase (EC 3.2.1.24); α-1,3-glucosidase (EC 3.2.1.84); and others. |
|  | GH32 | 1993,66 ± 41,97 | 1919 ± 51,42 | 2736,66 ± 43,32 | 1641,33 ± 133,49 | L+ *vs* C (p-value : 0.0194) | invertase (EC 3.2.1.26); endo-inulinase (EC 3.2.1.7); β-2,6-fructan 6-levanbiohydrolase (EC 3.2.1.64); endo-levanase (EC 3.2.1.65); exo-inulinase (EC 3.2.1.80); and others |
|  | GH36 | 351,33 ± 6,88 | 334,33 ± 8,98 | 380,33 ± 7,44 | 386 ± 45 | ns | α-galactosidase (EC 3.2.1.22); α-N-acetylgalactosaminidase (EC 3.2.1.49); stachyose synthase (EC 2.4.1.67); raffinose synthase (EC 2.4.1.82) |
|  | GH37 | 657 ± 76,57 | 822,66 ± 31,37 | 773,33 ± 8,76 | 3577,33 ± 468,45 | ns | α, α -trehalase (EC 3.2.1.28). |
|  | GH38 | 91 ± 32,12 | 104 ± 12,66 | 368,33 ± 53,34 | 731,33 ± 121,02 | AL+ *vs* C (p-value : 0.0395) | α-mannosidase (EC 3.2.1.24); mannosyl-oligosaccharide α-1,2-mannosidase (EC 3.2.1.113); mannosyl-oligosaccharide α-1,3-1,6-mannosidase (EC 3.2.1.114); and others. |
|  | GH43_10 | 334,66 ± 23,72 | 796,66 ± 30,23 | 357,66 ± 11,31 | 747 ± 118,39 | ns |  |
|  | GH43_26 | 1072,33 ± 91,71 | 1634 ± 47,84 | 1132,66 ± 18,26 | 4405,66 ± 565,34 | AL+ *vs* C (p-value : 0.0395) | exo-α-1,5-L-arabinofuranosidase (EC 3.2.1.-) ; α-L-arabinofuranosidase (EC 3.2.1.55) |
|  | GH65 | 448,33 ± 30,14 | 345 ± 4,5 | 471 ± 63,17 | 1163,33 ± 108,89 | AL+ *vs* C (p-value : 0.0194) | α,α-trehalase (EC 3.2.1.28); maltose phosphorylase (EC 2.4.1.8); trehalose phosphorylase (EC 2.4.1.64); kojibiose phosphorylase (EC 2.4.1.230); trehalose-6-phosphate phosphorylase (EC 2.4.1.216); and others. |
|  | GH73 | 2449,66 ± 50,71 | 2691,33 ± 100,59 | 5221 ± 201,55 | 7603,66 ± 123,65 | AL+ *vs* C (p-value : 0.0194) | lysozyme (EC 3.2.1.17); mannosyl-glycoprotein endo-beta-N-acetylglucosaminidase (EC 3.2.1.96); and others. |
|  | GH78 | 351,66 ± 6,69 | 235 ± 8,96 | 81 ± 7,76 | 387 ± 45,56 | AL+ *vs* C (p-value : 0.0395) | α-L-rhamnosidase (EC 3.2.1.40); rhamnogalacturonan α-L-rhamnohydrolase (EC 3.2.1.174); L-Rhap-α-1,3-D-Apif -specific α-1,3-L-rhamnosidase (EC 3.2.1.-) |
|  | GH88 | 662,66 ± 28,89 | 974,33 ± 30,62 | 413,33 ± 7,62 | 1081 ± 154,54 | ns | d-4,5-unsaturated β-glucuronyl hydrolase (EC 3.2.1.-) |
|  | GH92 | 94 ± 32,71 | 106,33 ± 13,22 | 373 ± 55,05 | 736,66 ± 119,35 | A+ *vs* C (p-value : 0.0395) | mannosyl-oligosaccharide α-1,2-mannosidase (EC 3.2.1.113); mannosyl-oligosaccharide α-1,3-mannosidase (EC 3.2.1.-); mannosyl-oligosaccharide α-1,6-mannosidase (EC 3.2.1.-); and others. |
|  | GH53 | 351 ± 7,09 | 234,33 ± 8,98 | 80 ± 7,21 | 386,33 ± 44,75 | L+ *vs* C (p-value : 0.0395) | endo-β-1,4-galactanase (EC 3.2.1.89). |
|  | GH63 | 351,66 ± 7,75 | 235 ± 9,53 | 80,66 ± 7,42 | 386,66 ± 44,68 | L+ *vs* C (p-value : 0.0461) | processing α-glucosidase (EC 3.2.1.106); α-1,3-glucosidase (EC 3.2.1.84); α-glucosidase (EC 3.2.1.20); and others. |
|  | GH68 | 351,33 ± 6,88 | 234,33 ± 8,98 | 80 ± 7,21 | 386 ± 45 | L+ *vs* C (p-value : 0.0395) | levansucrase (EC 2.4.1.10); β-fructofuranosidase (EC 3.2.1.26); inulosucrase (EC 2.4.1.9). |
| **Glycosyl transferase (GTs)** | GT0 | 354 ± 8 | 238,66 ± 10,71 | 85 ± 7 | 392,33 ± 45,1 | L+ *vs* C (p-value : 0.0383) | Glycosyltransferases not yet assigned to a family |
|  | GT1 | 18,33 ± 0,66 | 9,33 ± 1,76 | 10,66 ± 1,33 | 18,33 ± 2,02 | ns | UDP-glucuronosyltransferase (EC 2.4.1.17); zeatin O-β-xylosyltransferase (EC 2.4.2.40); 2-hydroxyacylsphingosine 1-β-galactosyltransferase (EC 2.4.1.45); and others. |
|  | GT2 | 75497,33 ± 6790,67 | 72695 ± 4652,58 | 65302 ± 1006,11 | 71633,66 ± 4001,45 | ns | cellulose synthase (EC 2.4.1.12); chitin synthase (EC 2.4.1.16); dolichyl-phosphate β-D-mannosyltransferase (EC 2.4.1.83); dolichyl-phosphate β-glucosyltransferase (EC 2.4.1.117); and others. |
|  | GT4 | 43160,66 ± 3911,22 | 38314,66 ± 2817,92 | 36300,33 ± 638 | 42946 ± 2821,61 | ns | sucrose synthase (EC 2.4.1.13) ; sucrose-phosphate synthase (EC 2.4.1.14) ; α-glucosyltransferase (EC 2.4.1.52) ; and others. |
|  | GT19 | 2237,33 ± 101 | 2466,33 ± 68,54 | 3439,66 ± 34,5 | 4684 ± 518,84 | A+ *vs* C (p-value : 0.0194) | Lipid-A-disaccharide synthase (EC 2.4.1.182). |
|  | GT20 | 646,33 ± 73,99 | 802 ± 31,04 | 748 ± 8,5 | 3458,33 ± 452,4 | ns | α,α-trehalose-phosphate synthase [UDP-forming] (EC 2.4.1.15); Glucosylglycerol-phosphate synthase (EC 2.4.1.213); trehalose-6-P phosphatase (EC 3.1.3.12); and others. |
|  | GT26 | 2183 ± 101,51 | 2416 ± 64,7 | 3379 ± 32,95 | 4597 ± 510 | A+ *vs* C (p-value : 0.0194) | UDP-ManNAcA: β-N-acetyl mannosaminuronyltransferase (EC 2.4.1.-); UDP-ManNAc: β-N-acetyl-mannosaminyltransferase (EC 2.4.1.-); and others. |
|  | GT28 | 2328 ± 74,51 | 2569,66 ± 73,58 | 3807,66 ± 76,33 | 5415,33 ± 400,75 | A+ *vs* C (p-value : 0.0194) | 1,2-diacylglycerol 3-β-galactosyltransferase (EC 2.4.1.46); 1,2-diacylglycerol 3-β-glucosyltransferase (EC 2.4.1.157);  and others. |
|  | GT30 | 2237 ± 100,68 | 2466 ± 68,41 | 3439,66 ± 34,5 | 4683,66 ± 518,86 | A+ *vs* C (p-value : 0.0194) | CMP-β-KDO: α-3-deoxy-D-manno-octulosonic-acid (KDO) transferase (EC 2.4.99.-). |
|  | GT35 | 1427 ± 109 | 1989,33 ± 55,32 | 1383,66 ± 19,46 | 1323 ± 117, 38 | ns | Glycogen or starch phosphorylase (EC 2.4.1.1). |
|  | GT5 | 1009 ± 86,81 | 1588,66 ± 51,34 | 1137,66 ± 20,57 | 4233,66 ± 544,7 | A+ *vs* C (p-value : 0.0395) | UDP-Glc : glycogen glucosyltransferase (EC 2.4.1.11); ADP-Glc: starch glucosyltransferase (EC 2.4.1.21); and others. |
|  | GT51 | 5433 ± 196,88 | 5532,33 ± 157,47 | 8847,33 ± 156,19 | 11252,66 ± 875,2 | ns | murein polymerase (EC 2.4.1.129). |
|  | GT8 | 1890,33 ± 57,64 | 1877,66 ± 46,38 | 4003,66 ± 96,85 | 2444 ± 01,211 | ns | lipopolysaccharide α-1,3-galactosyltransferase (EC 2.4.1.44);  UDP-Glc: (glucosyl)lipopolysaccharide α-1,2-glucosyltransferase (EC 2.4.1.-); lipopolysaccharide glucosyltransferase 1 (EC 2.4.1.58); and others. |
|  | GT84 | 352 ± 8,08 | 236,33 ± 9,52 | 81,33 ± 6,76 | 389 ± 45,56 | L+ *vs* C (p-value : 0.0395) | cyclic β-1,2-glucan synthase (EC 2.4.1.-); |
|  | GT56 | 1788 ± 132,5 | 2076 ± 61,8 | 3201,33 ± 25,27 | 4531,66 ± 507,89 | A+ *vs* C (p-value : 0.0194) | TDP-Fuc4NAc: lipid II Fuc4NAc transferase (EC 2.4.1.-) |
|  | GT9 | 5139,66 ± 160,66 | 5256,33 ± 125,85 | 6828 ± 82,39 | 8966,33 ± 960,66 | ns | lipopolysaccharide N-acetylglucosaminyltransferase (EC 2.4.1.56) ; heptosyltransferase (EC 2.4.-.-). |
|  | GT104 | 52,33 ± 2,02 | 47,66 ± 5,92 | 58±4,35 | 83,33 ± 8,87 | ns | dTDP-β-L-Rhap : arginine α-L-rhamnosyltransferase (EC 2.4.1.-) |
|  | GT73 | 310 ± 22,27 | 738,66 ± 27,72 | 331 ± 10,69 | 692,33 ± 109,01 | ns | CMP-β-KDO: α-3-deoxy-D-manno-octulosonic-acid (KDO) transferase (EC 2.4.99.-). |
|  | GT83 | 2128,33 ± 49,85 | 1994,66 ± 55,95 | 3274 ± 52,56 | 1630,66 ± 110,42 | L+ *vs* C (p-value : 0.0134) | undecaprenyl phosphate-α-L-Ara4N : 4-amino-4-deoxy-β-L-arabinosyltransferase (EC 2.4.2.43); and others. |
|  | GT101 | 95 ± 16,64 | 21,33 ± 8,41 | 38 ± 2,3 | 7 ± 1,15 | A+ *vs* C (p-value : 0.0132) | glucosyltransferase (EC 2.4.1.-) |
|  | GT25 | 95 ± 16,64 | 21,33 ± 8,41 | 38 ± 2,3 | 7 ± 1,15 | A+ *vs* C (p-value : 0.0132) | lipopolysaccharide β-1,4-galactosyltransferase (EC 2.4.1.-); β-1,3-glucosyltransferase (EC 2.4.1.-); β-1,2-glucosyltransferase (EC 2.4.1.-); and others. |
|  | GT108 | 351 ± 7,09 | 234,33 ± 8,98 | 80 ± 7,21 | 386 ± 45 | L+ *vs* C (p-value : 0.0395) |  |
|  | GT21 | 351,66 ± 7,75 | 235 ± 9,53 | 80,66 ± 7,42 | 386,66 ± 44,68 | L+ *vs* C (p-value : 0.0461) | UDP-Glc: ceramide β-glucosyltransferase (EC 2.4.1.80). |
|  | GT81 | 351 ± 7,09 | 234,33 ± 8,98 | 80 ± 7,21 | 386 ± 45 | L+ *vs* C (p-value : 0.0395) | NDP-Glc: glucosyl-3-phosphoglycerate synthase (EC 2.4.1.-); NDP-Man: mannosyl-3-phosphoglycerate synthase (EC 2.4.1.-); |
| **Carbohydrate Binding Module (CBMs)** | CBM5 | 1376.33 ± 26,672 | 924 ± 37.07 | 558.33 ± 33,7 | 1511.33 ± 171.83 | L+ *vs* C (p-value : 0.0395) | Found in bacterial enzymes. Chitin-binding function. |
|  | CBM48 | 1687,66 ± 108,89 | 2218,33 ± 59,73 | 1456,33 ± 17,83 | 5411,33 ± 674,58 | L+ *vs* C (p-value : 0.0134) | Glycogen-binding function. Also found in the β subunit (glycogen-binding) of AMP-activated protein kinases (AMPK) |
|  | CBM50 | 7571 ± 140,28 | 8170 ± 227,01 | 11793,33 ± 381,44 | 18778 ± 934,97 | A+ *vs* C (p-value : 0.0194) | Modules of approx. 50 residues found attached to various enzymes from families GH18, GH19, GH23, GH24, GH25 and GH73, i.e. enzymes cleaving either chitin or peptidoglycan. |
|  | CBM34 | 695 ± 53,5 | 1407,33 ± 62,88 | 1365,33 ± 29,4 | 4491 ± 385,03 | A+ *vs* C (p-value : 0.0134) | Granular starch-binding function; |
|  | CBM73 | 351 ± 7,09 | 234,33 ± 8,98 | 80 ± 7,21 | 386,33 ± 44,75 |  | Chitin-binding function; |
| **Carbohydrates Esterases (CEs)** | CE11 | 2236,66 ± 100,78 | 2466 ± 68,41 | 3439,33 ± 34,58 | 4683,66 ± 518,86 | A+ *vs* C (p-value : 0.0194) | UDP-3-0-acyl N-acetylglucosamine deacetylase (EC 3.5.1.108). |
|  | CE4 | 390,66 ± 9,52 | 283,66 ± 16,37 | 136,66 ± 4,09 | 466,66 ± 54,6 | L+ *vs* C (p-value : 0.0194) | acetyl xylan esterase (EC 3.1.1.72) ;  chitin deacetylase (EC 3.5.1.41); chitooligosaccharide deacetylase (EC 3.5.1.-); and others. |
|  | CE8 | 954,66 ± 86,76 | 1538,33 ± 48,03 | 1077,33 ± 19,19 | 4146,33 ± 536,24 | A+ *vs* C (p-value : 0.0395) | pectin methylesterase (EC 3.1.1.11). |
|  | CE9 | 2544,66 ± 86,07 | 2670 ± 75,83 | 4281,66 ± 84,36 | 5472,33 ± 391,32 | A+ *vs* C (p-value : 0.0279) | N-acetylglucosamine 6-phosphate deacetylase (EC 3.5.1.25) |
| **Enzymes with Auxiliary Activities (AAs)** | AA10 | 982 ± 19,65 | 682 ± 22,6 | 2655,33 ± 83,88 | 1146,33 ± 148,73 | AL+ *vs* L+  (p-value : 0.0134) | Copper-dependent lytic polysaccharide monooxygenases (LPMOs) acting on chitin and cellulose. |
| **Polysaccharides Lyases (PLs)** | PL22_1 | 454 ± 9,07 | 303 ± 11,59 | 104,66 ± 9,06 | 500,33 ± 57,81 | L+ *vs* C (p-value : 0.0395) | oligogalacturonate lyase / oligogalacturonide lyase (EC 4.2.2.6) |
|  | PL9_1 | 454 ± 9,07 | 303 ± 11,59 | 104 ± 9,29 | 500 ± 58,05 | L+ *vs* C (p-value : 0.0395) | pectate lyase (EC 4.2.2.2); exopolygalacturonate lyase (EC 4.2.2.9); thiopeptidoglycan lyase (EC 4.2.2.-); rhamnogalacturonan endolyase (EC 4.2.2.23) |
|  | PL17_2 | 412,66 ± 8,21 | 275,66 ± 10,74 | 93,66 ± 8,64 | 454 ± 52,93 | L+ *vs* C (p-value : 0.0395) | oligoalginate lyase / exo-alginate lyase (EC 4.2.2.26);oligomannuronate lyase / exo-acting mannuronan lyase (EC 4.2.2.26);poly(β-mannuronate) lyase / M-specific alginate lyase (EC 4.2.2.3) |
|  | PL38 | 351 ± 7,09 | 234,33 ± 8,98 | 80 ± 7,21 | 386 ± 45 | L+ *vs* C (p-value : 0.0395) | endo-β-1,4-glucuronan lyase (EC 4.2.2.14) |
