# Supplementary material for: Probiotic consortium modulating the gut microbiota composition and function of sterile Mediterranean fruit flies
Source: Sci Rep. 2024 Jan 11;14:1058. doi: 10.1038/s41598-023-50679-z (PMC10784543; doi:10.1038/s41598-023-50679-z)
Supplement: Supplementary file 5 — Supplementary Legends. [file 41598_2023_50679_MOESM5_ESM.docx]

**Figure S1.** Comparison of microbial variations using the linear discriminant analysis effect size (LEfSe). Cladogram for taxonomic representation of significant differences between L+ (a), A+ (b), AL+ (c) colony and the control (p < 0.05). Histogram of the LDA scores for differentially abundant features between L+ (d), A+ (e), AL+ (f) colony and the control. The threshold on the logarithmic LDA score for discriminative features was set to 2.0. Differences are represented in the color of the most abundant taxa.

**Figure S2.** The linkage between bacterial taxa and functions. The circos plots showing the linkage between the most abundant bacterial species taxonomic groups (Relative abundance > 1) and their corresponding (a) Carbohydrate metabolism, (b) Lipid metabolism, (c) Amino acid metabolism, (d) Metabolism of cofactors and vitamins, (e) Xenobiotic biodegradation and metabolism, (f) Biosynthesis of other secondary metabolites, (g) Metabolism of other Amino acids, and (h) Metabolism of terpenoids and polyetides, most abundant degradation pathways (KEGG pathways at level 3).

**Supplementary tables legends**

**Table S1:** The relative abundance distribution of different taxonomic classification level of the gut bacterial communities of A+, AL+, L+ and C colonies of C. capitata

**Table S2:** The Mean relative abundance distribution of predicted KEGG pathways at level 1 of the gut bacterial communities of A+, AL+, L+ and C colonies of C. capitata

**Table S3:** The mean relative abundance distribution of predicted KEGG pathways at level 2 of the gut bacterial communities of A+, AL+, L+ and C colonies of C. capitata

**Table S4:** The mean relative abundance distribution of predicted KEGG pathways at level 3 of the gut bacterial communities of A+, AL+, L+ and C colonies of C. capitata.

**Table S5:** Abundance of Carbohydrate active enzymes in guts microbes of different C. capitata colonies
